# Prevalence of Elevated Blood Pressure and Its Relationship With Anthropometric Risk Factors in Students of a Pre-university Girls’ College in Bangalore: A Cross-Sectional Study

**DOI:** 10.7759/cureus.49774

**Published:** 2023-12-01

**Authors:** Nazeela Kozhisseri, Dinesh Rajaram, Pavithra Cheluvaraj

**Affiliations:** 1 Public Health, Taluk Head Quarters Hospital, Ponnani, Ponnani, IND; 2 Community Medicine, Ramaiah Medical College, Bangalore, IND

**Keywords:** waist-to-hip ratio (whr), body mass index (bmi), waist circumference, overweight, pre-university, prevalence, obesity, adolescent girls, pre-hypertension, hypertension

## Abstract

Background

There is a growing concern regarding elevated blood pressure in adolescence. Children and adolescents with high blood pressure are at risk for adult hypertension. Being overweight and obese are important risk factors for hypertension. This study aimed to determine the prevalence of elevated blood pressure and its association with anthropometric risk factors among students of a pre-university girls’ college.

Methodology

A cross-sectional study was conducted among 337 students at a pre-university girls’ college aged 15-19 years in urban Bangalore. A self-administered, semi-structured, pretested questionnaire collected the sociodemographic details, family history, and lifestyle. Height, weight, waist circumference (WC), and hip circumference were measured. Standard cut-off levels were used for body mass index (BMI), WC, waist-hip ratio (WHR), and waist-height ratio (WHtR). Resting blood pressure was determined using a digital blood pressure monitor. It was classified into normotensive, pre-hypertension (>90th to <95th percentile), and hypertension (>95th percentile). Data were analyzed using SPSS version 18 (SPSS Inc., Chicago, IL, USA). Pre-hypertension and hypertension were considered as having elevated blood pressure.

Results

The prevalence of pre-hypertension and hypertension was 21.4% (n = 72, 95% confidence interval (CI) = 17.0-25.7) and 9.8% (n = 33, 95% CI = 6.6-13.0), respectively. The prevalence of overweight was 20.2% (n = 68, 95% CI = 15.9-24.5) and obesity was 12.2% (n = 41, 95% CI = 8.7-15.7). WC, WHR, and WHtR were abnormal in 34.7% (n = 117, 95% CI = 29.6-39.8), 47.5% (n = 160, 95% CI = 42.1-52.8), and 45.7% (n = 154, 95% CI = 50.4-51.0), respectively. There was a statistically significant correlation between systolic blood pressure and BMI (p < 0.001), WC (p < 0.001), and WHtR (p < 0.001), as well as diastolic blood pressure and BMI (p < 0.001), WC (p = 0.008), and WHtR (p = 0.011). Statistically significant differences in mean BMI (p = 0.004), WC (p < 0.001), WHR (p = 0.007), and WHtR (p = 0.001) between normal, pre-hypertensive, and hypertensive students were also noted.

Conclusions

Pre-hypertension and hypertension are fundamental problems in pre-university girl students. With a similarly increased prevalence of obesity and other anthropometric risk factors, students must be aware of hypertension and its risk factors.

## Introduction

Non-communicable diseases (NCDs) are increasing globally. India is facing a dual burden of disease, with communicable diseases at one end and an increasing burden of NCDs on the other end [1]. NCDs are often preceded by multiple risk factors, most of which are amenable to primary prevention if detected early. The main modifiable risk factors contributing to the rise in the prevalence of NCDs are physical inactivity, unhealthy diet, smoking, and alcohol use [2].

Systemic hypertension is one of the most common NCDs. According to the World Health Organization (WHO), one in every four men and one in every five women have hypertension [3]. It can lead to heart, brain, and kidney complications. One of the significant risk factors for the development of hypertension is overweight and obesity [4]. As the risk factors are similar for both NCDs and overweight and obesity, a rise in the prevalence of both has been noted [5].

Currently, worldwide, the prevalence of systemic hypertension is low in children and adolescents. However, there is a growing concern that it is rising in this age group, ranging from 2% to 21.5%. According to a Centers for Disease Control and Prevention study, an estimated 1.3 million youth aged 12 to 19 have high blood pressure, with one in 25 youth having hypertension and one in 10 having elevated blood pressure globally [6]. India has the largest adolescent population in the world, with 253 million adolescents, and every fifth person is aged 10-19 years old [7]. Children and adolescents with high blood pressure are at risk for adult hypertension [8]. Although hypertension is more prevalent in men than women of young age, it is important to pay equal attention to the prevalence of hypertension in women [9]. Elevated blood pressure can impact women at different phases of life, especially during pregnancy. It is essential to assess the blood pressure status in adolescent girls to facilitate early behavioral changes. The present study was conducted to determine the prevalence of elevated blood pressure and its association with anthropometric risk factors among students of a pre-university girls’ college.

## Materials and methods

A cross-sectional study was conducted between July 2018 and September 2019 among 337 students of a private pre-university girls’ college in Bangalore after obtaining ethical clearance from the Ramaiah Medical College Institutional Review Board (approval number: SS-1/EC019/2017) and written informed consent and assent from the students chosen via convenience sampling. The students belonged to the age group of 15-19 years. Based on a study by Ranjani et al., a sample size of 337 was obtained by calculating a 21.5% prevalence of elevated blood pressure, with 5% alpha error, and 4.5% absolute precision [10]. All students present on the day of the study were included, and those on medications known to cause high blood pressure were excluded. Baseline information including sociodemographic details and the family history of study participants was collected using a semi-structured, pretested questionnaire. The participants’ socioeconomic status was classified based on the modified B G Prasad scale [11]. Anthropometric measurements such as height, weight, waist circumference (WC), and hip circumference were measured using calibrated instruments.

Height was measured using a non-stretchable measuring tape following standard protocols and rounded off to the nearest 0.5 cm. Weight was measured using a digital weighing machine following standard protocols and rounded off to the nearest 0.1 kg. Waist circumference was taken using a non-stretchable measuring tape at the midpoint between the top of the iliac crest and the lower margin of the last palpable rib in the midaxillary line. The tape was kept parallel to the ground. Measurement was rounded off to the nearest 0.1 cm. Hip measurement was taken using a non-stretchable measuring tape at the broadest portion of the buttocks with the tape parallel to the ground. The study participants were made to stand with arms at the sides, both feet together, and weight evenly spread on both feet. Measurement was rounded off to the nearest 0.1 cm. All measurements were done following the WHO STEPS guidelines.

Body mass index (BMI) was categorized based on the following cut-offs: BM1 ≥27 kg/m^2^ (≥95th percentile) and ≥23 kg/m^2^ (≥75th percentile) for obese and overweight, respectively, based on the Revised Indian Association of Pediatrics Growth Charts for Height, Weight, and Body Mass Index for 5- to 18-year-old Indian Children [12]. A WC of more than 80 cm, a waist-hip ratio (WHR) of more than 0.85, and a waist-height ratio (WHtR) of more than 0.5 were considered above average [13,14].

A single blood pressure measurement was taken using an OMRON HEM-7120 automatic blood pressure monitor. The blood pressure was recorded in a relaxed sitting posture on the right upper arm at heart level. Blood pressure was classified into normotensive, pre-hypertension (>90th to <95th percentile), and hypertension (>95th percentile) based on the Fourth Report on the Diagnosis, Evaluation, and Treatment of High Blood Pressure in Children and Adolescents [15]. For those aged 18 years and above, an adult cut-off was used to categorize blood pressure.

Operational definition

Values greater than 120/80 mmHg and less than 140/90 mmHg were considered pre-hypertension, and values greater than 140/90 mmHg were considered hypertension [16]. Those having pre-hypertension and hypertension were considered to be having elevated blood pressure.

Statistical analysis

Data were entered in Microsoft Excel (Microsoft Corp., Redmond, WA, USA) and analyzed using SPSS version 18.0 (SPSS Inc., Chicago, IL, USA). All quantitative variables were expressed in terms of mean and standard deviation or median and interquartile range. All categorical variables were expressed as percentages. Quantitative variables were tested for normal distribution using the Kolmogorov-Smirnov test. Differences between means of anthropometric variables in outcome groups were made using analysis of variance or t-tests. The difference between the median of those anthropometric variables (BMI), which did not follow normality, was assessed using the Kruskal-Wallis test. The prevalence of pre-hypertension and hypertension were calculated as percentages. The chi-square test was used to assess the association between risk factors and outcome variables. Fisher’s exact test was used to test the association between variables that had an expected value of less than five in the cells. Pearson’s correlation coefficient was computed among various quantitative variables and tested for statistical significance.

## Results

This study was done among 337 students of a girls’ pre-university college in Bangalore. The prevalence of pre-hypertension and hypertension was 21.4% (n = 72, 95% confidence interval (CI) = 17.0-25.7) and 9.8% (n = 33, 95% CI = 6.6-13.0), respectively. The prevalence of overweight was 20.2% (n = 68, 95% CI = 15.9-24.5) and obesity was 12.2% (n = 41, 95% CI = 8.7-15.7). WC, WHR, and WHtR were abnormal in 34.7% (n = 117, 95% CI = 29.6-39.8), 47.5% (n = 160, 95% CI = 42.1-52.8), and 45.7% (n = 154, 95% CI = 50.4 -51.0), respectively. The mean BMI, WC, WHR, and WHtR of study participants were 21.40 kg/m2 (SD = 4.40), 75 cm (SD = 11.09), 0.83 (SD = 0.06), and 0.49 (SD = 0.07), respectively. Sociodemographic characteristics and family history of hypertension are described in Table 1.

**Table 1:** Sociodemographic characteristics of study participants (n = 337). *: according to the modified B G Prasad scale.

	Frequency	Percentage
Age (completed years)		
15	14	4.2
16	135	40.1
17	151	44.8
18	34	10.1
19	3	0.9
Religion		
Hindu	312	92.6
Muslim	16	4.7
Christian	7	2.1
Others	2	0.6
Socio economic status^*^		
I	12	3.6
II	142	42.1
III	147	43.6
IV	36	10.7
V	0	0
Family history of hypertension		
Present	72	21.4
Absent	195	57.9
Do not know	70	20.8
Total	337	100

A statistically significant correlation was found between BMI, WC, WHtR, and systolic blood pressure. Diastolic blood pressure was also significantly correlated with BMI, WC, and WHtR (Table 2).

**Table 2:** Pearson’s correlation of blood pressure with selected anthropometric risk factors (n = 337).

		Anthropometric variables			
		Body mass index	Waist circumference	Waist-hip ratio	Waist-height ratio
Systolic blood pressure (mmHg)	Pearson’s correlation	0.262	0.262	0.101	0.234
	Significance	0.000	0.000	0.065	0.000
Diastolic blood pressure (mmHg)	Pearson’s correlation	0.191	0.145	0.061	0.139
	Significance	0.000	0.008	0.267	0.011

Significant differences were noted in the mean values of selected anthropometric measures across the normal, pre-hypertensive, and hypertensive groups (Table 3).

**Table 3:** Mean and standard deviation of anthropometric variables of study participants (n = 337). *: median and interquartile range.

	Normal	Pre-hypertension	Hypertension	P-value
Body mass index*	20 (18.2–23.2)	21.7 (18.35–24.35)	23.5 (19.25–27)	0.004
Waist circumference	74.5 (10.15)	76.82 (11.69)	82.36 (13.72)	0.000
Waist-hip ratio	0.835 (0.065)	0.837 (0.066)	0.874 (0.066)	0.007
Waist-height ratio	0.48 (0.07)	0.49 (0.07)	0.54 (0.08)	0.001

A statistically significant association was observed between age (p = 0.369), religion (p = 0.355), socioeconomic status (p = 0.441), family history of hypertension (p = 0.077), BMI (p = 0.067), WC (p = 0.85), waist-hip ratio (p = 0.159), and waist-height ratio (p = 0.489) with hypertension or pre-hypertension.

## Discussion

In this study of 337 students from pre-university girls’ colleges in Bangalore, the prevalence of pre-hypertension and hypertension was 21.4% and 9.8%, respectively. Lower prevalence compared to our study was found in studies by Buch et al. in Surat and Sabapathy et al. in rural Bangalore [17,18] Studies by Abolfoutoh et al. in Egypt and Gyamfi et al. in Ghana also observed a lower prevalence of hypertension [19,20]. However, the latter study noted a higher prevalence of pre-hypertension [18]. Qaddumi et al. in Palestine found a higher prevalence of hypertension and pre-hypertension [21]. In National Family Health Survey 4 (NFHS-4), the prevalence of hypertension in women aged 15-19 years was 2.8%, which was much lower than that reported in this study; however, the cut-off taken for hypertension in NFHS-4 was blood pressure more than 140/90 mmHg [22].

The possible risk factors for the study participants to have a higher prevalence of hypertension are overweight and obesity. The prevalence of overweight was 20.2%, and obesity was 12.2%. WC, WHR, and WHtR were abnormal in 34.7%,47.5%, and 45.7%, respectively. Possible mechanisms of obesity leading to hypertension are insulin resistance, sodium retention, increased sympathetic nervous system activity, activation of renin-angiotensin-aldosterone, and altered vascular function [23].

In this study, there was a significant correlation between BMI, WC, WHtR, and systolic blood pressure, as well as with diastolic blood pressure. A correlation between systolic and diastolic blood pressure was noted with WHtR and BMI by Gyamfi et al. [20] Similar to our study, Gyamfi et al. did not find a correlation with WHR [20]. There was a significant difference in mean values of anthropometric variables across normotensive, pre-hypertensive, and hypertensive groups. The median BMI in the normotensive group was 20, in the pre-hypertensive group was 21.7, and in the hypertensive group was 23.5. The difference in this median BMI was statistically significant.

The mean WC in the normotensive group was 74.5, in the pre-hypertensive group was 76.82, and in the hypertensive group was 82.36. The difference in mean WC was statistically significant. The mean WHR in the normotensive group was 0.835, in the pre-hypertensive group was 0.837, and in the hypertensive group was 0.874. The difference in the mean WHR was statistically significant.

The mean WHtR in the normotensive group was 0.48, in the pre-hypertensive group was 0.49, and in the hypertensive group was 0.54. The difference in mean WHtR was statistically significant. According to a systematic review conducted by Kelishadi et al., irrespective of the anthropometric measurement used, there was a higher risk for hypertension in an adolescent with abdominal obesity [24].

There was no statistically significant association between age, religion, socioeconomic status, family history of hypertension, BMI, WC, WHR, and WHtR with pre-hypertension and hypertension. In a study by Abolfoutoh et al., BMI, WHR, WC, and WHtR showed significant associations with hypertension [19]. Significant association was also observed with a family history of hypertension, which was not observed in our study. Few previous studies also did not observe a significant association between a family history of hypertension and hypertension in study participants [17,20]. As parents’ blood pressure could not be ascertained, the actual prevalence of hypertension in the family may be underestimated.

The exact prevalence of hypertension is difficult to ascertain as studies use different age groups, criteria, sampling methods, and different instruments for measuring anthropometry and blood pressure. As this study was an institution-based study with convenience sampling, generalization of the results to a larger population is impossible. Serial or ambulatory blood pressure monitoring is recommended to rule out white-coat and masked hypertension and reduce overdiagnosis [15,25]. However, further follow-up of students was not logistically feasible as the students were from different parts of the city and were in the institution only for one to two years.

The present study had a large sample that helped in providing precise estimates. The same instrument was used throughout the study with regular calibration. Blood pressure was measured with study participants at ease. Studies concentrating on adolescent girls are fewer. Hence, this study helps bridge the knowledge gap in this gender and age group.

Limitations

This study did not consider other factors that influence the development of hypertension, such as physical inactivity, stress, salt intake, and other dietary factors, which might have affected the results. A single reading of blood pressure was taken in our study as it was not feasible to take two readings as per standard protocol due to not getting permission from the college authorities for multiple interactions with the students which could have influenced the results. Convenience sampling was used which could affect the results. Therefore, a more extensive community-based study with a longitudinal blood pressure recording is needed in this age group.

## Conclusions

Pre-hypertension and hypertension are fundamental problems in pre-university girl students. Awareness of hypertension and modifiable risk factors must be generated among children and adolescents. School health in pre-university or senior secondary schools is covered under Ayushman Bharat through Rashtriya Bal Swasthya Karyakram (RBSK) in India, which currently does not stress on NCDs, including hypertension. It is recommended that age-appropriate health promotion activities and periodic measurements of anthropometric variables and blood pressure are incorporated into RBSK at the secondary school level to prevent the development of NCDs.

## References

[1] Chauhan S, Kumar S, Patel R, Simon DJ, Kumari A (2022). Burden of communicable and non-communicable diseases-related inequalities among older adults in India: a study based on LASI survey. BMC Geriatr.

[2] Forouzanfar MH, Afshin A, Alexander LT (2023). Status of non-communicable diseases (NCDs) in India. Lancet.

[3] (2023). Hypertension. https://www.who.int/news-room/fact-sheets/detail/hypertension.

[4] Falkner B (2010). Hypertension in children and adolescents: epidemiology and natural history. Pediatr Nephrol.

[5] (2017). Worldwide trends in body-mass index, underweight, overweight, and obesity from 1975 to 2016: a pooled analysis of 2416 population-based measurement studies in 128·9 million children, adolescents, and adults. Lancet.

[6] (2023). High blood pressure in kids and teens. Internet].[cited.

[7] (2023). Adolescent development and participation. https://www.unicef.org/india/what-we-do/adolescent-development-participation.

[8] Juhola J, Magnussen CG, Berenson GS (2013). Combined effects of child and adult elevated blood pressure on subclinical atherosclerosis: the International Childhood Cardiovascular Cohort Consortium. Circulation.

[9] Ahmad A, Oparil S (2017). Hypertension in women: recent advances and lingering questions. Hypertension.

[10] Jayashri B, Aarti S, Vinod A (2015). Pre-hypertension and hypertension and its determinants among school adolescents of rural area of Indore - a cross-sectional study. Natl J Community Med.

[11] Pentapati SS, Debnath DJ (2023). Updated BG Prasad's classification for the year 2022. J Family Med Prim Care.

[12] (2017). Revised IAP growth charts for height, weight and body mass index for 5- to 18-year-old Indian children. http://indianpediatrics.net/jan2015/jan-47-55.htm.

[13] Geneva. Waist Circumference and Waist-Hip Ratio (2019). Waist circumference and waist-hip ratio: report of a WHO expert consultation. https://www.who.int/publications/i/item/9789241501491.

[14] Yoo EG (2016). Waist-to-height ratio as a screening tool for obesity and cardiometabolic risk. Korean J Pediatr.

[15] Falkner B, Daniels SR, Flynn JT (2004). The fourth report addresses diagnosing, evaluating, and treating high blood pressure in children and adolescents. Paediatrics.

[16] (2017). American College of Cardiology. 2017 guideline for the prevention, detection, evaluation, and management of high blood pressure in adults. https://www.acc.org/~/media/Non-Clinical/Files-PDFs-Excel-MS-Word-etc/Guidelines/2018/Guidelines-Made-Simple-Tool-2018-Cholesterol.pdf.

[17] Buch N, Goyal JP, Kumar N, Parmar I, Shah VB, Charan J (2011). Prevalence of hypertension in school going children of Surat city, Western India. J Cardiovasc Dis Res.

[18] Sabapathy S, Nagaraju BA, Bhanuprakash CN (2017). Prevalence of childhood hypertension and pre-hypertension in school-going children of Bangalore rural district: a cross-sectional study. Int J Contemp Pediatr.

[19] Abolfotouh MA, Sallam SA, Mohammed MS, Loutfy AA, Hasab AA (2011). Prevalence of elevated blood pressure and association with obesity in Egyptian school adolescents. Int J Hypertens.

[20] Bosu WK, Bosu DK (2021). Prevalence, awareness and control of hypertension in Ghana: a systematic review and meta-analysis. PLoS One.

[21] Qaddumi J, Holm M, Alkhawaldeh A (2016). Prevalence of hypertension and pre-hypertension among secondary school students. Int J Adv Nurs Stud.

[22] International Institute for Population Sciences (IIPS) (2017). National Family Health Survey (NFHS-4). https://www.google.com/url?sa=t&rct=j&q=&esrc=s&source=web&cd=&ved=2ahUKEwiupIbM9O2CAxUixTgGHX59C38QFnoECA0QAQ&url=https%3A%2F%2Fdhsprogram.com%2Fpubs%2Fpdf%2Ffr339%2Ffr339.pdf&usg=AOvVaw0NcBPpQHQQMkBFtXvHzeuL&opi=89978449.

[23] Kotchen TA (2010). Obesity-related hypertension: epidemiology, pathophysiology, and clinical management. Am J Hypertens.

[24] Kelishadi R, Mirmoghtadaee P, Najafi H, Keikha M (2015). Systematic review on the association of abdominal obesity in children and adolescents with cardio-metabolic risk factors. J Res Med Sci.

[25] Ashraf M, Irshad M, Parry NA (2023). Pediatric hypertension: an updated review. Clin Hypertens.

